# Adeno-Associated Viral Vectors as Versatile Tools for Neurological Disorders: Focus on Delivery Routes and Therapeutic Perspectives

**DOI:** 10.3390/biomedicines10040746

**Published:** 2022-03-23

**Authors:** Ana Fajardo-Serrano, Alberto J. Rico, Elvira Roda, Adriana Honrubia, Sandra Arrieta, Goiaz Ariznabarreta, Julia Chocarro, Elena Lorenzo-Ramos, Alvaro Pejenaute, Alfonso Vázquez, José Luis Lanciego

**Affiliations:** 1 Centro de Investigación Médica Aplicada (CIMA), Department of Neuroscience, Universidad de Navarra, 31008 Pamplona, Spain; ajrico@unav.es (A.J.R.); eroda@unav.es (E.R.); ahonrubia@unav.es (A.H.); sarrietae@unav.es (S.A.); garizna@unav.es (G.A.); jchocarrog@unav.es (J.C.); erl@unav.es (E.L.-R.); apejenaute@unav.es (A.P.); 2 Centro de Investigación Biomédica en Red de Enfermedades Neurodegenerativas (CiberNed), 23038 Madrid, Spain; 3 Instituto de Investigación Sanitaria de Navarra (IdiSNA), 31008 Pamplona, Spain; alfonso.vazquez.miguez@cfnavarra.es; 4 Department of Neurosurgery, Servicio Navarro de Salud, Complejo Hospitalario de Navarra, 31008 Pamplona, Spain

**Keywords:** AAV, gene therapy, disease-modifying therapeutics, neuroprotection, precision medicine

## Abstract

It is without doubt that the gene therapy field is currently in the spotlight for the development of new therapeutics targeting unmet medical needs. Thus, considering the gene therapy scenario, neurological diseases in general and neurodegenerative disorders in particular are emerging as the most appealing choices for new therapeutic arrivals intended to slow down, stop, or even revert the natural progressive course that characterizes most of these devastating neurodegenerative processes. Since an extensive coverage of all available literature is not feasible in practical terms, here emphasis was made in providing some advice to beginners in the field with a narrow focus on elucidating the best delivery route available for fulfilling any given AAV-based therapeutic approach. Furthermore, it is worth nothing that the number of ongoing clinical trials is increasing at a breath-taking speed. Accordingly, a landscape view of preclinical and clinical initiatives is also provided here in an attempt to best illustrate what is ongoing in this quickly expanding field.

## 1. Introduction

Adeno-associated viral vectors (AAVs) are members of *Dependoparvovirus* in the *Parvoviridae* family. AAVs require co-infection with adenovirus (Ad), baculovirus, or herpes simplex virus to complete the replication cycle, with Ad being the natural helper virus in clinical isolates. An AAV without helper can integrate into the genome, but cannot be propagated by itself, which makes it a safer choice for gene therapy [1]. AAVs were first identified by electron microscopy [2], and they are formed by an icosahedral capsid carrying a single-stranded linear DNA genome that contains two open-reading frames encoding for Rep (40, 52, 68, and 78) involved in replication and integration, the capsid (Cap), three structural proteins (VP1, VP2, and VP3), and a small viral cofactor for assembly-activating protein [3]. Rep-independent recombinant AAVs are used for gene therapy purposes, in order to avoid the preference of integration of Rep proteins into the AAVS1 site inside of Ch19 [1,4].

The origin of AAV-based technologies started when the plasmid clone of wild AAV showed infective behavior when transfected into human cells after Ad helper co-infection [5]. This discovery demonstrated the feasibility of a transient and persistent expression for a marker gene lasting for 6 months or more with AAVs [6,7].

When designing any given AAV-based experiment in the central nervous system (CNS), there are two important prerequisites to be taken into consideration at first glance: (i) choosing the best suited AAV, with a proper balance between the AAV serotype and its expected neurotropism, and (ii) selection of the promoter driving the desired transgene expression (e.g., either ubiquitous or cell-specific). The most commonly used promoters for CNS applications are CAG, CBA, JeT, GusB, and EF1, among others [8]. Different promoters may have different potencies when driving transgene expression. Indeed, for late-stage preclinical developments, ensuring a proper balance between efficacy and safety often represents a critical issue. The use of small-sized promoters is a convenient strategy in order to leave enough cargo space when accommodating large-sized genes [9,10]. Once the choice of best AAV serotype and promoter is made, the most critical decision to be reached before pushing forward any given successful therapeutic approach is to elucidate the most adequate route for AAV delivery, as described below.

## 2. AAV Delivery Routes

When coming to design any AAV-based therapeutics, the choice of the delivery route represents the most critical decision for achieving the best balance of safety, efficacy, and target engagement. In other words, evidence supporting that any given therapeutic product enters the brain and reaches the right target in a concentration high enough to be efficient needs to be provided. In addition to delivery routes targeting neurosensory organs such as the eye or the cochlea, the most frequently used approaches for CNS applications can be broadly categorized into (i) intraparenchymal, (ii) intra-CSF (intrathecal or lumbar administration, intracisternal, and intracerebroventricular), (iii) intravenous, (iv) intramuscular, and (v) intranasal [11]. Final choice for delivery also needs to be tailored taking into consideration the CNS disorder to be dealing with. In recent years, the gene therapy field has witnessed an exponential increase in initiatives rising up to unprecedented levels, particularly when dealing with CNS applications, as summarized in Table 1.

The most commonly used routes for AAV delivery in the brain are intraparenchymal and intra-CSF (lumbar, intracisternal, or intracerebroventricular). Although less commonly used, a subpial delivery route has also been reported elsewhere [91,92]. Other ways to cope with CNS disorders bypassing the blood–brain barrier (BBB) are intranasal delivery [93], systemic eye delivery, and ear delivery [68,69,70,73,81,83,84]. In the case of disorders engaging motor neurons of the spinal cord, intramuscular delivery can also be viewed as a feasible approach [53,54,63,65].

## 3. Intraparenchymal Deliveries

Intraparenchymal AAV delivery requires stereotaxic surgery, a procedure where a needle or cannula is inserted directly into the desired target area, as defined with three coordinates (e.g., rostrocaudal, mediolateral, and dorsoventral coordinates). By delivering the viral vector this focused way, a high transduction efficiency is expected; therefore, the intraparenchymal delivery is the choice most frequently used in the treatment of brain disorders such as Alzheimer disease (AD), Huntington disease (HD) (see Table 1 and Table 2), or Parkinson disease (PD) [11]. When translating preclinical research toward clinical uses, the use of pressurized convection-enhanced delivery (CED) is the procedure most often used [94,95,96]. Compared to any other available delivery route, the intraparenchymal approach holds several advantages, such as (i) high transduction efficacy within the target region, (ii) reduced amounts of AAV needed (both in terms of total delivered volume and titration), (iii) BBB bypassing, (iv) little concern—if any—when dealing with neutralizing antibodies, and (v) off-target effects (e.g., transduction of peripheral organs) very unlikely.

Regarding intraparenchymal deliveries, the recent availability of AAV capsid variants engineered to enhance retrograde spread of the encoded transgene also represents an appealing choice. Among others, AAV2-retro [97], AAV-TT [98], and AAV-MNM008 [99] are well suited for multiple transduction of neurons innervating the injected site.

## 4. Intra-CSF Deliveries

Intra-CSF AAV deliveries collectively represent another feasible way for viral vector administration. This administration is less invasive than intraparenchymal delivery. Furthermore, compared to intravenous administration, a reduced immune response together with fewer off-target effects in peripheral organs is expected. It can be achieved through lumbar puncture, cisterna magna injection, or administration into the lateral ventricles [100] (Table 1). However, a potential toxic effect at the level of the dorsal root ganglia needs to be taken into consideration [101]. Although this delivery route has its own inherent advantages, vector dilution and the limited penetration/transduction in deep brain structures collectively represent important limiting factors that need to be properly balanced before pushing forward any therapeutic development [11]. In this regard, it is worth noting that the CSF volume is replaced five times per day in humans, and the pattern of CSF circulation indeed needs to be properly understood when tailoring therapeutic uses. In our experience, intra-CSF deliveries of AAV resulted in highly variable patterns of neuronal transduction throughout the cerebral cortex, only affording a desired consistent pattern when dealing with efficient transduction of neurons in the spinal cord.

## 5. Intravenous Delivery Routes

Intravenous AAV deliveries have been widely used in the past (see Table 1). Although some AAV serotypes—AAV9 in particular—have been reported to be efficient when transducing the CNS upon systemic delivery, some concerns still remain regarding BBB passage. Highest efficacy rates were obtained in newborn animals, whereas there is a limited BBB penetration in adult animals. In an attempt to circumvent this limitation, years ago Viviana Gradinaru and Benjamin Deverman developed the AAV9 variant known as AAV9-PHP.B and AAV9-PHP.eB (making reference to “enhanced B”, introduced later on), a capsid variant specifically designed for enhancing BBB bypass [102]. Although initial results afforded an impressive performance for AAV9-PHP.B in C57BL6 mice, some limitations in terms of BBB penetrance were reported later on when using different strains of mice, as well as in NHPs [103,104]. Regardless of BBB passage, main limitations inherent to systemic deliveries can be broadly summarized as (i) need for high volume of AAV to be injected, with high titration levels, (ii) undesired off-target effects, in particular potential liver toxicity, and (iii) limited CNS transduction, at least when relying on most of the currently available AAV capsid variants.

## 6. AAV Delivery in Sensory Organs

**Figure 1:**
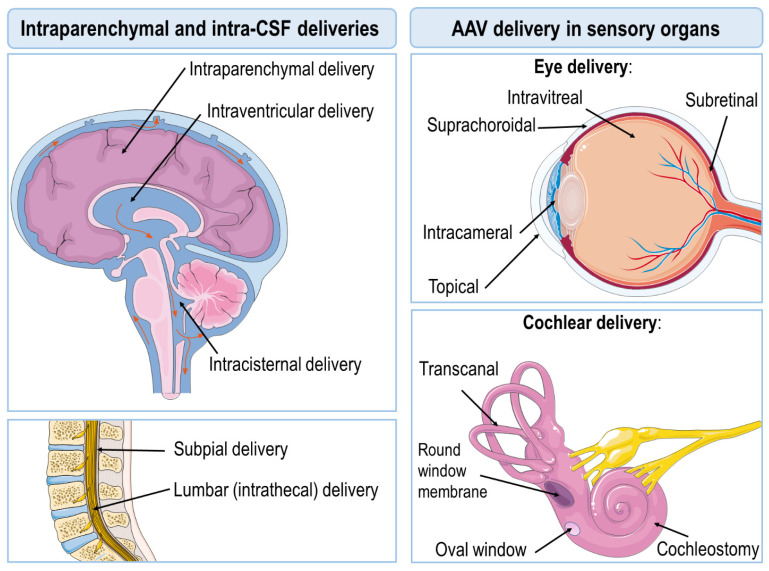
Illustration of most commonly used AAV delivery routes. For CNS diseases (e.g., Parkinson, Alzheimer, Huntington), the intraparenchymal administration of viral particles is by far the strategy most commonly used, followed by intra-CSF administration (intraventricular, intracisternal, and intrathecal). Several ongoing gene therapy studies are focused on targeting blindness and deafness disorders, and, in these scenarios, eye delivery (e.g., subretinal, intravitreal, intracameral, etc.) and ear delivery (e.g., cochleostomy or RWM) have proven preclinical success.

Direct AAV delivery into the eye currently represents a good example of preclinical experiments translated to several ongoing clinical trials. There are several different delivery options, such as (i) subretinal, (ii) intravitreal, (iii) intracameral, (iv) subchroidal, or (v) topical (Figure 1). Both the subretinal and the intravitreal choices are those most commonly used [70,75], somewhat predictable considering the isolation and compartmentalization of the eye and the specificity of an injection in these areas. When considering targeting the inner ear, AAV delivery can be achieved through cochlear injection, transcanal administration, oval window, or the row window membrane (RWM) (Table 1 and Figure 1). Unlike AAV eye delivery, the ear delivery of AAVs has still not yet entered into clinical practice, although a number of promising preclinical studies are currently ongoing (Table 1).

## 7. AAV-Mediated Therapeutic Uses: The Path to the Clinical Scenario

The use of AAVs for the treatment of CNS disorders exemplifies translation of preclinical evidence toward clinical trials, beginning with pioneer experiences [105,106], up to a quickly growing list of clinical trials. Indeed, a broad majority of the ongoing AAV clinical trials are targeting several neurological diseases. Among the different AAV serotypes available, AAV2 and AAV9 rank as the most commonly used within the context of PD [11]. AAV2 undergoes anterograde axonal transport in rat and non-human primate brain [107,108], while AAV9 shows both anterograde and retrograde transport [109]. The use of AAV2 often is the main option in the case of AD, eye delivery-related diseases, and other neurological disease as Batten disease. On the other hand, AAV9 is the most popular choice for neuromuscular dystrophies or atrophies such as ALS or SMA.

When considering PD under a simplistic view as a basal ganglia-related disorder primarily affecting the nigrostriatal pathway, the most rationale scenario implies an intraparenchymal delivery route administering a given therapeutic AAV either into the substantia nigra pars compacta (SNc) or into the striatum [11,110,111]. Considering AD as a whole-brain disorder, intraparenchymal, intracisternal, or intrathecal administrations are the options most commonly used. Lastly, diseases such as SMA are usually approached through either intravenous or intramuscular injections (Table 1).

Ongoing gene therapy clinical trials for PD can be broadly categorized on the basis of the chosen target: (i) dopamine-related, (ii) neurotrophic factors, (iii) neuromodulators, and (iv) specific genetic mutations. Dopamine-related approaches take advantage of AAVs coding for l-aromatic acid decarboxylase (AADC), the enzyme converting levodopa into dopamine [112,113,114]. Neurotrophic factors such as GDNF or NRTN have also been introduced into the clinical path [115,116,117,118,119], with GDNF AAV-based therapies currently witnessing a revival. Regarding, neuromodulation, some clinical trials have been carried out using the enzyme glutamic acid decarboxylase (GAD) [120,121,122,123,124], with the purpose of switching the functional activity of the STN from excitation to inhibition. Lastly, targeting particular genetic mutations in disease-related genes has recently opened a completely new scenario. This is the case of glucocerebrosidase (GCase), a lysosomal enzyme encoded by the GBA1 gene [125]. When going this way, promising results were obtained in several different preclinical studies carried out in mice and in NHP [126,127,128].

Similarly to PD, gene therapy ongoing clinical trials in the AD field can also be categorized on the basis of the selected target: (i) neurotrophic factors from the GDNF family, brain-derived neurotrophic factor (BDNF), and beta-nerve growth factor (NGF), (ii) neuromodulators such as GAD, and (iii) specific mutations, particularly in apolipoprotein E (APOE).

Within the field of motor-related neurological disorders, SMA is a good example of ongoing clinical trials with AAVs. When considering SMA, the survival of motor neuron (SMN) is the preferred choice (Table 2). Treatments intended to overexpress cytotoxic T cell GalNAc transferase (GALGT2) in skeletal muscles for the purpose of inhibiting the development of muscular dystrophy have been explored in mice [129]. Moreover, the use of human alpha-sarcoglycan (hαSG) has shown efficacy for treatment of muscular dystrophies. Despite several preclinical attempts made for testing AAV-related therapies for the treatment of ALS, ongoing clinical trials challenging this devastating disorder are still lacking. A single dose of a DNA-based gene therapy (AVXS-101 or Zolgensma^®^) has been approved for the clinical treatment of SMA type 1. Although the beneficial effect of this treatment is clear, increases in AST and ALT liver enzymes have been reported. Resulting from this therapy, life expectancy increased for children enrolled in the trial. The clinical results suggested persistence of the transgene activity in the treated patients [130,131,132]; however, thrombotic microangiopathy (TMA) has been reported as an undesired side effect sometimes observed. The expected beneficial effect for gene therapy-based treatments targeting genetic disorders needs to be properly balanced with issues such as liver toxicity, vascular injury, and neurotoxicity. 

The clinical trials against HD are usually focused on the specific mutation of the huntingtin protein (Htt). Htt is the main cause of the disease, and it is involved in axonal transport, related to vesicles and microtubules. Currently, there are two ongoing clinical trials on early stages (Table 2).

Vision loss and retinal degeneration processes are appealing choices for AAV therapeutics, considering that peripheral sensory organs such as the eye are easily accessible and, therefore, fully approachable through a direct AAV delivery. Luxturna^®^ was the first gene therapy treatment receiving FDA approval (NCT00999609). Intravitreal and subretinal injection are useful choices when targeting disorders such as Leber’s congenital amaurosis, retinosis pigmentosa, choroideremia, achromatopsia, retinal neurodegeneration, retinal dystrophy, retinoschisis, and age-related macular degeneration (Table 2). 

## 8. Conclusions

The field of gene therapy has witnessed the arrival of new viral serotypes and capsids which have contributed to bringing AAV-based therapies closer than ever to the clinical scenario. More arrivals to the field have been constantly incorporated at a breathtaking speed. Considering gene therapy overall, main expectancies for therapeutic success are currently represented by CNS applications. Although the best is yet to come, for the very first time, the potential success of disease-modifying treatments is achievable. When implementing AAV-based therapeutics for neurological considerations, there are at least three important items to be properly balanced: (i) biosafety, (ii) selection of the most appropriate target gene, and (iii) disease-tailored delivery route. Furthermore, rare disorders are creating a completely new scenario for gene therapy application; indeed, it is worth nothing that roughly half of the lysosomal storage disorders have a neurological impact, most often related to neurodegenerative pathologies. Lastly, incoming advanced novel therapeutics such as gene therapies are demanding a clear regulatory scenario, to properly preserve patient and pharmaceutical expectations, reaching an adequate balance across all engaged stakeholders. Accordingly, recent advice issued by the FDA is a good step forward in this direction, clarifying underlying rules and regulations within the adequate framework. 

## Figures and Tables

**Table 1:** Summary of selected ongoing initiatives which have been available in recent years for different CNS disorders approached by with AAV-based therapeutics, such as Alzheimer disease (AD), Huntington disease (HD), amyotrophic lateral sclerosis (ALS), and spinal muscular atrophy (SMA), as well as either vision- or hearing-related diseases. Abbreviations: Aβ (β-amyloid), APOE (apolipoprotein E), shIRS1 (short hairpin RNA against insulin receptor substrate 1), NTR (neurotrophin receptor), CCL2 (chemokine L2), ECE (endothelin-converting enzyme), NGF (nerve growth factor), scFv (semisynthetic anti-Aβ antibody), PHF1 (monoclonar antibody against TAU), IL-10 (interleukin-10), BDNF (brain-derived neurotrophic factor), GDNF (glial cell-derived neurotrophic factor), HTT (huntingtin protein), SIRT3 (mitochondrial protein deacetylase), XBP1 (X-box binding protein 1), ZNF10 (zinc finger protein 10), SREBP2 (sterol regulatory element-binding protein 2), AAT (α-1 antitrypsin), SOD1 (superoxide dismutase 1), HGF (hepatocyte growth factor), hIGF1 (insulin-like growth factor 1), DOK7 (tyrosine kinase 7), GLT1 (glutamate transporter 1), NMJ (neuromuscular junction), TGF-β1 (transforming growth factor beta 1), CAD180 (calreticulin anti-angiogenic domain), SYNE4 (spectrin repeat containing nuclear envelope family member 4), XIAP (X-linked inhibitor of apoptosis).

Disease	Delivery Routes	Target	Species	AAV Serotype	References
Alzheimer	Intraparenchymal	Aβ	Mice	AAV1	[12]
	Intraparenchymal	APOE2	Mice	AAV9 and AArh10	[13]
	Intraparenchymal	shIRS1 (IRS1: neuroprotective role)	Rats	AAV2/DJ8	[14]
	Intraparenchymal	CCL2 (diffuse amyloid plaques)	Mice	AAV1/2	[15]
	Intraparenchymal	ECE (protease involved in Aβ degradation)	Mice	AAV5	[16]
	Intraparenchymal	NGF (improving cholinergic activity)	Rats	AAV2 and AAV5	[17]
	Intraparenchymal	NGF	Mice	CERE-110 (AAV2)	[18]
	Intraparenchymal	PHF1 (anti-phospho-TAU antibody)	Mice	AAVrh10	[19]
	Intraparenchymal	CascFv59 (anti-Aβ antibody)	Mice	AAV2	[20]
	Intraparenchymal	IL-10 (inhibition of proinflammatory cytokines)	Mice	AAV1	[21]
	Intramuscular and intravenous	GFP	Mice	AAV9, exo-AAV9 (IM) and AAV8 (IV)	[22]
	Intramuscular	scFv (anti-Aβ antibody)	Mice	AAV1	[23]
	Intramuscular	P75NTR (protective against Aβ)	Mice	AAV8	[24]
	Intracerebroventricular	GFP	Mice	AAV1, AAV5, AAV8, AAV9, AAV2-BR1 and AAV2-PHP.eB	[25]
Huntington	Intraparenchymal	82Q (mutant Htt)	Rats	AAV2	[26]
	Intraparenchymal	BDNF and GDNF	Rats	AAV2	[27]
	Intraparenchymal	CRISPR/Cas9 (Htt)	Mice	AAV1	[28]
	Intraparenchymal	SIRT3 (protective against oxidative and mitochondrial stress)	Mice	AAV-DJ	[29]
	Intraparenchymal	XBP1 (involved in the splicing events of Htt)	Mice	AAV2	[30]
	Intraparenchymal	mRNA or siRNA (Htt)	Mice	AAV9	[31]
	Intraparenchymal	iRNA (Htt)	Mice	AAV8	[32]
	Intraparenchymal	Exon1-Q138 mHtt and Exon1-Q17 wildtype Htt	Mice	AAV9	[33]
	Intraparenchymal	Human KRAB domain from KOX1 (ZNF10); ZNF10 represses mutant Htt expression	Mice	AAV9	[34]
	Intraparenchymal	GFP	Rats	AAV1, AAV2 and AAV5	[35]
	Intraparenchymal	GDNF (neurturin)	Mice	AAV8	[36]
	Intraparenchymal	miHDS1 (Htt)	Mice	AAV1	[37]
	Intraparenchymal	SREBP2 (to reverse synaptic defects in Huntington disease)	Mice	AAV5	[38]
	Intraparenchymal	siRNA (Htt)	Sheep	AAV serotype not disclosed	[39]
	Intravenous	iRNA (Htt)	Mice	AAV1	[40]
	Intramuscular and intravenous	shRNA (AAT)	Mice	AAV8 (IV) and AAV6 (IM)	[41]
	Intrathecal	miRNA based on endogenous mir155 backbone (Htt)	Sheep	AAV9	[42]
Amyotrophic lateral sclerosis	Intraparenchymal and intramuscular	GFP	Mice	AAV1, AAV2, AAV5, AAV6, AAV7, AAV8	[43]
	Intravenous and intracisternal	SOD1	Mice	AAVrh10	[44]
	Intravenous	IGF1	Mice	AAV9	[45]
	Intravenous	GDNF	Rat	AAV9	[46]
	Intracerebroventricular	GFP	Mice	AAV9	[47]
	Intramuscular	HGF in SOD1 model	Mice	AAV6	[48]
	Intramuscular	hIGF1 in SOD1model	Mice	AAV9	[49]
	Intramuscular	GDNF	Mice	AAV2	[50]
	Intramuscular	GDNF	Mice	AAV2	[51]
	Intramuscular	GFP	Mice	AAV1, AAV5, AAV8 and AAV9	[52]
	Intramuscular	SOD1	Mice	AAV6	[53]
	Intramuscular	IGF1 and GDNF	Mice	AAV2	[54]
	Intramuscular	IGF1	Mice	AAV9	[55]
	Intrathecal	GLT1 overexpression in SOD1 animal model	Mice	AAV8	[56]
	Intrathecal	SOD1	Mice	AAV9	[57]
	Intracisternal	C9orf72 hexanucleotide repeat expansions (generates neuropathology)	Mice	AAV9	[58]
Spinal muscular atrophy	Intracerebroventricular and intraperitoneal	GFP	Mice	AAV9	[59]
	Intracerebroventricular	SMN1 (gene replacement strategy)	Mice	AAV9	[60]
	Intracerebroventricular (mice) and intracisternal (pigs and NHP)	hSMN1	Mice, Pigs, and NHPs	AAV9	[61]
	Intracerebroventricular and intravenous	SMN1	Mice	AAV9	[62]
	Intramuscular	DOK7 (tuning down disease severity)	Mice	AAV9	[63]
	Intravenous	SMN transgene	Piglets and NHPs	AAVhu68	[64]
	Intramuscular	GFP	Mice	AAV9	[65]
	Intrathecal	SMN2 (to rescue the SMA model)	Mice	AAV9	[66]
	Intracisternal	miRNA	Mice	AAVrh10	[67]
Vision disorders	Subconjuntival	GFP	Mice	AAV2, AAV6 and AAV8	[68]
	Intravenous	CRISPR/Cas9 (retinitis pigmentosa)	Mice	AAV2, AAV6 and AAV8	[69]
	Subretinal	TGF-β1 (retinitis pigmentosa)	Mice	AAV8	[70]
		GFP	Mice and NHPs	AAV7m8 and AAV8BP2	[71]
		GFP	Mice	AAV8, AAV9. AAV-PHP.B, AAV-PHP.eB	[72]
		GFP	Mice and pigs	AAV8	[73]
	Retinal	CRISPR/Cas9 (retinal editing)	Mice	AAV2 and AAV7	[74]
	Intravitreal	CAD180 (endogenous inhibitor of angiogenesis) retinal neovascularization (RNV)	Mice	AAV2	[75]
		GFP	NHPs	AAV2	[76]
		GFP	Mice and NHPs	AAV2	[77]
		GFP	Mice	AAV2, AAV5, AAV8 and AAV9	[78]
Hearing disorders	Cochlear	CRISPR/Cas9 (gene editing)	Mice	AAV2	[79]
		SYNE4 (to rescue in a deafness model)	Mice	AAV9-PHP.B	[80]
		GFP	Mice	AAV2, AAV6, AAV8, AAV/Anc80L65	[81]
		GFP	Mice and guinea pigs	AAV2, AAV9 and Anc80L65	[82]
	Canalastomy (inner ear cells)	GFP	Mice	AAV1, AAV2, AAV6.2, AAV8, AAV9, AAVrh.39, AAVrh.43 and Anc80L65	[83]
		CRISPR/Cas9 (GFP, Biodistribution)	Mice	AAV8	[84]
	Round window membrane	XIAP against Cisplatin (chemotherapeutic agent)	Mice	AAV2	[85]
		GFP	Mice and NHPs	AAV9-PHP.B	[86]
		Harmonin-a1 and harmonin-b1 (To rescue Usher syndrome type 1c)	Mice	AAV1 and AAV/Anc80L65	[87]
		GFP	Mice	AAV1 and exo-AAV1	[88]
		GFP	Mice	AAV2/DJ, AAV2/DJ8, AAV2-PHP.B	[89]
	Utricle (inner and outer cells)	GFP	Mice	AAV9-PHP-B, Anc80L65 and AAV2.7m8	[90]

**Table 2:** AAV-based clinical trials for neurological disorders with AAV for PD, AD, HD, SMA and blindness related diseases. (http://www.genetherapynet.com/clinical-trials.html; last access: 10 February 2022). Abbreviations: hTERT (active telomerase), CM (cisterna magna), STN (subthalamic nucleus), NBM (nucleus basalis of Meynert), TH (thalamus), AADC (aromatic l-amino acid decarboxylase), GDNF (glial cell-derived neurotrophic factor), GAD (glutamic acid decarboxylase), NRTN (neurturin), GBA (lysosomal enzyme glucocerebrosidase), APOE (apolipoprotein E), NGF (nerve growth factor), BDNF (brain-derived neurotrophic factor), HTT (huntingtin), RPGR (retinitis pigmentosa GTPase regulator), MCO-I (multi-characteristic opsin I), ND4 (NADH-ubiquinone oxidoreductase chain 4, IP (intraparenchymal), ICV (intracerebroventricular), IV (intravenous), IT (intrathecal), IC (intracisternal), IM (intramuscular), IVT (intravitreal), SR (subretinal), REP1 (Rab escort protein 1), RPE (retinal pigment epithelium), MERTK (proto-oncogene tyrosine kinase MER), PDE6B (phosphodiesterase 6B), RS1 (retinoschisin 1), Ab (antibody), VEGF (vascular endothelial growth factor), CNGA3 (cyclic nucleotide-gated cation channel alpha-3), CNGB3 (cyclic nucleotide-gated cation channel beta-3).

Disease		Clinical Trial	Duration	Phase	Target	AAV Serotype	Delivery Routes	Status	Company References
Parkinson		NCT01973543	2013–2020	I	AADC	AAV2	IP in the Putamen	Completed	[112] University of California
		NCT02418598	2015–2018	I/II	AADC	AAV2	IP in the Putamen	Terminated (another clinical study for regulatory approval is planned)	[113] Jichi Medical University
		NCT03065192	2017–2021	I	AADC01	AAV2	IP in the Putamen	Active, not recruiting	*Neurocrine Biosciences*
		NCT03562494	2018–2022	II	AADC02	AAV2	IP	Active, not recruiting	[114] *Voyager Therapeutics* (*Neurocrine Biosciences*)
		NCT03733496	2018–2026	IV	AADC01	AAV2	IP in the Putamen	Enrolling, by invitation	[112,133,134] *Voyager Therapeutics* (*Neurocrine Biosciences*)
		NCT04167540	2020–2022	I	GDNF	AAV2	IP in the Putamen	Recruiting	*Ask Bio* (*formerly Brain Neurotherapy Bio, Inc.*)
		NCT01621581	2013–2022	I	GDNF	AAV2	IP in the Putamen	Completed	[114,115,116,117] *National Institute of Neurological Disorders and Stroke*
		NCT00643890	2008–2010	II	GAD	AAV2	IP in the STN	Terminated (due to financial reasons)	[120,121,122,123] *Neurologix, Inc*.
		NCT00195143	2003–2005	I	GAD	AAV2	IP in the STN	Completed	[121,122,123,124] *Neurologix, Inc.*
		NCT01301573	2011–2012	IV	GAD	AAV2	IP in the STN	Terminated (due to financial reasons)	*Neurologix, Inc.*
		NCT00252850	2005–2007	I	NRTN	CERE-120 (AAV2)	IP in the Putamen	Completed	[118] *Ceregene*
		NCT00985517	2009–2017	I/II	NRTN	CERE-120 (AAV2)	IP in the Putamen	Completed	[119] *Sangamo Therapeutics*
		NCT00400634	2006–2008	II	NRTN	CERE-120 (AAV2)	IP in the Putamen	Completed	[118] *Ceregene*
		NCT04127578	2020–2027	I/II	GBA1	AAV9	IC in the CM	Recruiting	*Prevail Therapeutics*
Alzheimer		NCT03634007	2019–2023	I	APOE2	AAVrh.10h	IC in the CM	Recruiting	*Lexeo Therapeutics*
		NCT04133454	2019–2021	I	hTERT	N.A.	IV and IT	The status was recruiting; currently unknown	*Libella Gene Therapeutics*
		NCT00087789	2004–2010	I	NGF	CERE-110 (AAV2)	IP in the NBM	Completed	*Ceregene*
		NCT00876863	2008–2015	II	NGF	CERE-110 (AAV2)	IP in the NBM	Completed	[135] *Sangamo Therapeutics*
		NCT05040217	2021–2025	I	BDNF	AAV2	IP	Recruiting	[136,137]
Huntington’s disease		NCT04885114	2021–2024	I	miHtt	AAV1	IP in the Putamen and TH	Withdrawn (novel AAV that may enable IV delivery)	*Voyager Therapeutics*
		NCT04120493	2019–2026	I/II	miHtt	AAV5	IP in the striatum	Recruiting	[138] *UniQure Biopharma B.V.*
Spinal muscular atrophy		NCT03306277	2017–2019	III	SMN	AAV9	IV	Completed	[139] *Novartis Gene Therapies*
		NCT04042025	2020–2035	IV	SMN	AAV9	IV	Enrolling by invitation	*Novartis Gene Therapies*
		NCT03837184	2019–2021	III	SMN	AAV9	IV	Completed	*Novartis Gene Therapies*
		NCT02122952	2014–2017	I	AVXS-101	AAV9	IV	Completed	[140,141]
		NCT03461289	2018–2020	III	SMN	AAV9	IV	Completed	*Novartis Gene Therapies*
		NCT03381729	2017–2024	I	SMN	AAV9	IT	Completed	*Novartis Gene Therapies*
Vision-related diseases	Leber’s congenital amaurosis	NCT02781480	2016–2018	I/II	RPE65	AAV2/5	SR	Completed	*MeiraGTx UK II*
		NCT01496040	2011–2014	I/II	RPE65	AAV2/4	SR	Completed	*Nantes University Hospital*
		NCT00516477	2007–2018	I	RPE65	AAV2	SR	Completed	*Spark Therapeutics*
		NCT00999609	2012–2029	III	RPE65	AAV2	SR	Active, not recruiting	[142,143] *Spark Therapeutics*
		NCT00821340	2016–2017	I	RPE65	AAV2	SR	Completed	[144,145] *Hadassah Medical Organization*
		NCT00481546	2007–2026	I	RPE65	AAV2	SR	Active, not recruiting	[146,147] *University of Pennsylvania*
		NCT02946879	2016–2023	I/II	RPE65	AAV2/5	SR	Recruiting	*MeiraGTx UK II*
		NCT00749957	2009–2017	I/II	RPE65	AAV2	SR	Completed	[144,148] *Applied Genetic Technologies Corp*
		NCT02161380	2014–2023	I	ND4	AAV2	IVT	Active, not recruiting	[149] *University of Miami*
		NCT02652767	2016–2019	III	ND4	AAV2/2	IVT	Completed	[150] *GenSight Biologics*
		NCT02652780	2016–2018	III	ND4	AAV2/2	IVT	Completed	[150] *GenSight Biologics*
		NCT03153293	2017–2025	II/III	ND4	AAV2	IVT	Active, not recruiting	[151,152]
	Retinitis pigmentosa	NCT01482195	2011–2019	I	MERTK	AAV2	SR	Completed	[153] *King Khaled Eye Specialist Hospital*
		NCT03116113	2017–2020	III	BIIB112 (RPGR)	AAV8	SR	Enrolling by invitation	[154] *NightstaRx, Biogen Company*
		NCT03252847	2017–2020	I/II	RPGR	AAV2/5	SR	Completed	*MeiraGTx UK II*
		NCT03326336	2018–2025	I/II	GS030-DP	AAV2.7m8	IVT	Recruiting	*GenSight Biologics*
		NCT04919473	2019–2020	I/II	vMCO-I	AAV2	IVT	Completed	*Nanoscope Therapeutics*
		NCT03328130	2017–2026	I/II	PDE6B	AAV2/5	SR	Recruiting	[155,156] *Horama*
		NCT04945772	2021–2023	II	vMCO-010	AAV2	IVT	Recruiting	*Nanoscope Therapeutics*
		NCT04850118	2021–2029	II/III	RPGR	AAV2	SR	Not yet recruiting	*Applied Genetic Technologies*
		NCT03316560	2018–2026	I/II	RPGR	AAV2	SR	Recruiting	*Applied Genetic Technologies*
		NCT04312672	2019–2023	I/II	RPGR	AAV2	SR	Recruiting	*MeiraGTx UK II*
	Retinitis pigmentosa/choroideremia	NCT03584165	2018–2027	III	BIIB111 (REP1) and BIIB112 (RPGR)	AAV2 and AAV8	SR	Enrolling by invitation	*NightstaRx, Biogen Company*
	Choroideremia	NCT02161380	2011–2017	I/II	REP1	AAV2	SR	Active, not recruiting	[157,158,159,160] *University of Oxford*
		NCT02553135	2015–2018	III	REP1	AAV2	SR	Enrolling by invitation	[161] *University of Miami*
		NCT03507686	2018–2022	III	BIIB111 (REP1)	AAV2	SR	Enrolling by invitation	[161] *NightstaRx, Biogen Company*
		NCT02077361	2015–2025	III	REP1	AAV2	SR	Enrolling by invitation	[147,162] *University of Alberta*
		NCT02671539	2016–2018	III	REP1	AAV2	SR	Enrolling by invitation	[163] *STZ eyetrial*
		NCT03496012	2017–2020	III	BIIB111 (REP1)	AAV2	SR	Enrolling by invitation	[161] *NightstaRx, Biogen Company*
		NCT02341807	2015–2022	I/II	REP1	AAV2	SR	Active, not recruiting	*Spark Therapeutics*
		NCT02407678	2016–2021	III	REP1	AAV2	SR	Enrolling by invitation	*University of Oxford*
	Achromatopsia	NCT03758404	2019–2021	I/II	CNGA3	AAV2/8	SR	Completed	*MeiraGTx UK II*
		NCT02935517	2017–2025	I/II	CNGA3	AAV2	SR	Recruiting	[164] *Applied Genetic Technologies Corp*
		NCT02599922	2016–2025	I/II	hCNGB3	AAV2	SR	Recruiting	[165] *Applied Genetic Technologies Corp*
		NCT03001310	2017–2019	I/II	CNGB3	AAV2/8	SR	Completed	*MeiraGTx UK II*
		NCT03278873	2017–2024	I/II	CNGB3 & CNGA3	AAV2/8	SR	Active, not recruiting	*MeiraGTx UK II*
	Retinal degeneration	NCT00643747	2007–2014	I/II	RPE65	AAV2/2	SR	Completed	[145] *University College, London*
	Retinal dystrophy	NCT04516369	2020–2026	III	RPE65	AAV2	SR	Active, not recruiting	*Novartis Pharmaceuticals*
	Retinoschisis	NCT02416622	2015–2023	I/II	RS1	AAV2	IVT	Active, not recruiting	*Applied Genetic Technologies*
	Age-related macular degeneration	NCT03748784	2018–2022	I	aflibercept	AAV.7m8	IVT	Active, not recruiting	*Adverum Biotechnologies*
		NCT04645212	2020–2025	IV	aflibercept	AAV.7m8	IVT	Enrolling by invitation	*Adverum Biotechnologies*
		NCT03066258	2017–2021	I/II	RGX-314 (Ab against VEGF)	AAV8	SR	Active, not recruiting	*Regenxbio*
		NCT04832724	2021–2022	II	RGX-314	AAV8	SR	Recruiting	*Regenxbio*
	Diabetic macular edema/ diabetic retinopathy	NCT04418427	2020–2022	II	aflibercept	AAV.7m8	IVT	Active, not recruiting	*Adverum Biotechnologies*

## Data Availability

Data reported here are available from authors upon reasonable request.
